# Development of a new drug candidate for the inhibition of Lassa virus glycoprotein and nucleoprotein by modification of evodiamine as promising therapeutic agents

**DOI:** 10.3389/fmicb.2023.1206872

**Published:** 2023-07-11

**Authors:** Shopnil Akash, Javiera Baeza, Sajjat Mahmood, Nobendu Mukerjee, Vetriselvan Subramaniyan, Md. Rezaul Islam, Gaurav Gupta, Vinibha Rajakumari, Suresh V. Chinni, Gobinath Ramachawolran, Fayez M. Saleh, Ghadeer M. Albadrani, Amany A. Sayed, Mohamed M. Abdel-Daim

**Affiliations:** ^1^Department of Pharmacy, Faculty of Allied Health Sciences, Daffodil International, University, Dhaka, Bangladesh; ^2^Center for Bioinformatics and Molecular Simulation, Universidad de Talca, Talca, Chile; ^3^Millennium Nucleus of Ion Channels-Associated Diseases (MiNICAD), Universidad de Chile, Santiago, Chile; ^4^Department of Microbiology, Jagannath University, Dhaka, Bangladesh; ^5^Department of Microbiology, West Bengal State University, West Bengal, Kolkata, India; ^6^Department of Health Sciences, Novel Global Community Educational Foundation, Hebersham, NSW, Australia; ^7^Pharmacology Unit, Jeffrey Cheah School of Medicine and Health Sciences, MONASH University, Jalan Lagoon Selatan, Bandar Sunway, Selangor, Malaysia; ^8^Center for Transdisciplinary Research, Department of Pharmacology, Saveetha Institute of Medical and Technical Sciences, Saveetha Dental College and Hospital, Saveetha University, Chennai, Tamil Nadu, India; ^9^School of Pharmacy, Suresh Gyan Vihar University, Jagatpura, Jaipur, India; ^10^Uttaranchal Institute of Pharmaceutical Sciences, Uttaranchal University, Dehradun, India; ^11^Faculty of Foundation, MAHSA University, Selangor, Malaysia; ^12^Department of Biochemistry, Faculty of Medicine, Bioscience, and Nursing, MAHSA University, Selangor, Malaysia; ^13^Department of Periodontics, Saveetha Dental College and Hospitals, Saveetha Institute of Medical and Technical Sciences, Saveetha University, Chennai, India; ^14^Department of Foundation, RCSI & UCD Malaysia Campus, Pulau Pinang, Malaysia; ^15^Department of Medical Microbiology, Faculty of Medicine, University of Tabuk, Tabuk, Saudi Arabia; ^16^Department of Biology, College of Science, Princess Nourah bint Abdulrahman University, Riyadh, Saudi Arabia; ^17^Department of Zoology, Faculty of Science, Cairo University, Giza, Egypt; ^18^Department of Pharmaceutical Sciences, Pharmacy Program, Batterjee Medical College, Jeddah, Saudi Arabia; ^19^Department of Pharmacology, Faculty of Veterinary Medicine, Suez Canal University, Ismailia, Egypt

**Keywords:** Lassa fever virus, emerging viral infections, drug discovery, ADMET, molecular docking, molecular dynamics simulation, evodiamine

## Abstract

The Lassa virus (LASV), an RNA virus prevalent in West and Central Africa, causes severe hemorrhagic fever with a high fatality rate. However, no FDA-approved treatments or vaccines exist. Two crucial proteins, LASV glycoprotein and nucleoprotein, play vital roles in pathogenesis and are potential therapeutic targets. As effective treatments for many emerging infections remain elusive, cutting-edge drug development approaches are essential, such as identifying molecular targets, screening lead molecules, and repurposing existing drugs. Bioinformatics and computational biology expedite drug discovery pipelines, using data science to identify targets, predict structures, and model interactions. These techniques also facilitate screening leads with optimal drug-like properties, reducing time, cost, and complexities associated with traditional drug development. Researchers have employed advanced computational drug design methods such as molecular docking, pharmacokinetics, drug-likeness, and molecular dynamics simulation to investigate evodiamine derivatives as potential LASV inhibitors. The results revealed remarkable binding affinities, with many outperforming standard compounds. Additionally, molecular active simulation data suggest stability when bound to target receptors. These promising findings indicate that evodiamine derivatives may offer superior pharmacokinetics and drug-likeness properties, serving as a valuable resource for professionals developing synthetic drugs to combat the Lassa virus.

## 1. Introduction

The Lassa virus (LASV), an Arenavirus family member, is responsible for causing hemorrhagic fever and multiple organ failure. Transmission to humans occurs through rodents and human-to-human contact and is considered endemic in several West African countries, including Sierra Leone, Liberia, Guinea, and Nigeria (Agbonlahor et al., 2021). The first documented case of the Lassa virus dates back to 1969 in Nigeria, and annual reports suggest an incidence of 100,000–300,000 cases, resulting in approximately 5,000 fatalities (CFDCA Prevention, 2022). However, these numbers are rough estimates due to regional variations in Lassa fever surveillance. In Sierra Leone and Liberia, Lassa fever accounts for 10–16% of hospitalized patients with the infection (CFDCA Prevention, 2022). From week 1 to week 52 of 2022, at least 1,067 LASV cases were diagnosed across 112 Local Government Areas (LGAs) and 27 states of the Nigerian Federation, with 189 fatalities resulting from the disease. According to the latest report from the Nigeria Center for Disease Control (NCDC), 8,202 cases were reported from 26 December 2022 to 1 January 2023, affecting more than 63 healthcare personnel (Vanguard, 2023).

LASV's genome is a single-stranded, bipartite ribonucleic acid (RNA), lacking an arenavirus's typical negative-strand coding configuration (Andersen et al., 2015). The spherical Lassa virus ranges from 70 to 150 nm in size and features a glycoprotein envelope with T-shaped spikes measuring 7–10 nm on its surface (Ogbu et al., 2007).

Although the virus's pathophysiology is not fully understood, research has shown that it primarily targets endothelial and antigen-presenting cells, particularly dendritic cells, and upon entering the human body, the Lassa virus infects most tissues, initially affecting the mucosa, intestine, lungs, and urinary system, followed by the vascular system (Mahanty et al., 2003; Rojek et al., 2008). LASV RNA genome is responsible for encoding a few translational products such as highly glycosylated membrane glycoprotein (MGP), RNA polymerase, a matrix protein, and a nucleoprotein (NP). Among these genomic products membrane, glycoprotein plays a crucial role in viral attachment and fusion through endothelial cell surface (Meyer et al., 2002). Lassa fever membrane glycoprotein consists of two subunits (GP1 and GP2), where GP1 is responsible for receptor binding and GP2 plays a significant role in cell membrane fusion (Lenz et al., 2001; Igonet et al., 2011; Borenstein-Katz et al., 2019). MGP incorporates with the α-dystroglycan receptor of the extracellular matrix and initiates LASV entry into the host cell (Bowen et al., 2000). The pathogen replicates intracellularly by utilizing L-polymerase and nucleocapsid proteins. After that, nucleocapsid proteins synthesize both mRNAs and antigenomic RNAs that are responsible for evading the host immune system (Yun and Walker, 2012). The entire pathophysiology of the Lassa fever virus depends on the successful attachment of the virus with endothelial cells. However, the Lassa virus glycoprotein spike (PDB ID 5FT2) was considered a putative drug target in our study to inhibit viral attachment with host endothelial cells (Li et al., 2016).

However, the Lassa virus NP is critical for both transcription and RNA replication since it encloses viral genomic RNAs into ribonucleoprotein (RNP) complexes (Hass et al., 2004). Though the exact mechanism of NP involvement in the pathogen's pathophysiology is poorly understood, it has been shown in prior research that the NP of the Lassa virus plays an essential role in viral RNA synthesis and host immune system suppression by actively suppressing type I interferon (IFN) (Mart-nez-Sobrido et al., 2006; Martnez-Sobrido et al., 2007). Generalized immune suppression in the infected host is correlated with severe arenavirus infections, including fatal Lassa cases, which indicates that the NP of the Lassa virus is a key element in Lassa fever pathogenesis (Baize et al., 2009). Therefore, Lassa virus nucleoprotein (PDB ID: 3MX5) was also considered as a potential drug target for our following study (Qi et al., 2010).

**Figure 1:**
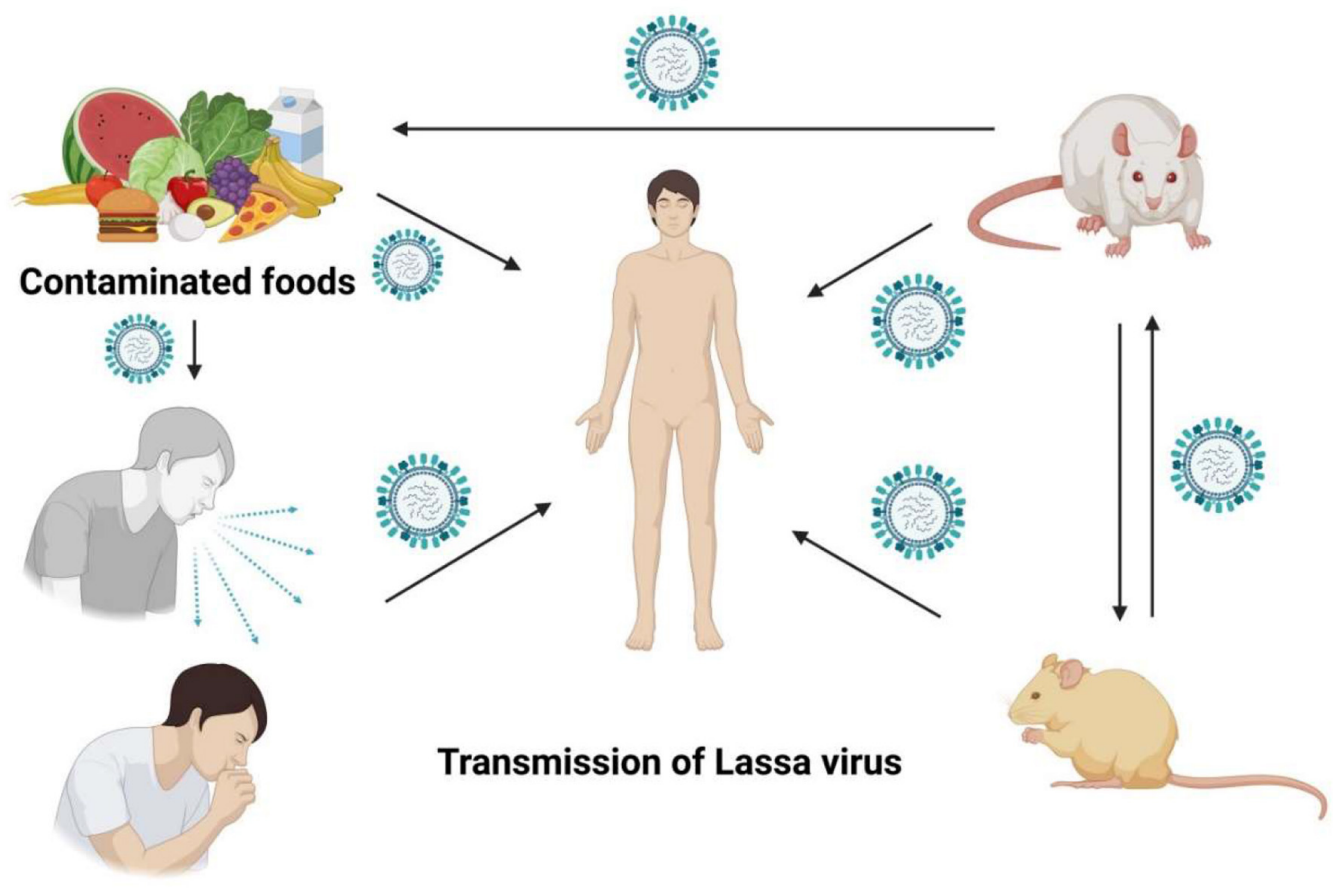
Unveiling the stealthy spread of the Lassa virus, a tale of rodents, humans, and unexpected encounters.

The “multimammate rat,” a rodent species, serves as the primary reservoir or host for the Lassa virus. The two predominant modes of Lassa virus transmission to humans are ingestion and inhalation (Tewogbola and Aung, 2020; CFDCA Prevention, 2022). Mastomys rats excrete the virus in their urine and droppings, and direct exposure to these materials—such as handling contaminated objects, consuming tainted food, or contacting open wounds or sores—can result in infection. Lassa virus typically infects humans upon contact with the urine or feces of infected Mastomys rats or through direct contact with the blood, urine, feces, or other bodily secretions of an individual suffering from Lassa fever (McCormick and Fisher-Hoch, 2002; Atkin et al., 2009). Infection can occur when humans come into contact with contaminated rat excrement or when they capture and consume the rodents as food. Lassa fever can be contracted from an infected individual, although this is relatively rare (Asogun et al., 2019). Blood, saliva, urine, and semen are some of the bodily fluids that can spread infection from person to person, occurring in both household and healthcare settings. Those in close contact with the infected individuals are generally only exposed to the patient's symptoms; however, a patient can excrete the virus through their semen for up to 3 months and in their urine for 3–9 weeks, following the onset of their illness (Azeez-Akande, 2016) (Figure 1).

In a world where the menacing Lassa virus poses a deadly threat, we find ourselves with limited therapeutic options and no FDA-approved drugs or vaccines for treating Lassa fever (Baral et al., 2020). The pressing need for effective antiviral treatments has driven researchers to explore new frontiers, turning to computational studies in search of potential drug candidates. Meanwhile, numerous biological actions of evodiamine, including anti-inflammatory, anti-viral anti-tumor, blood pressure reduction, and immunological modulation, have been reported (Dai et al., 2012; Gavaraskar et al., 2015; Yang F. et al., 2017; Zhang et al., 2022). Evodiamine, a naturally occurring indole alkaloid derived from the traditional medicinal plant *Evodia rutaecarpa*, has been increasingly recognized in recent years for its various pharmacological effects, including anti-inflammatory, anti-cancer, and anti-obesity properties, to name a few. Intriguingly, some studies have also shown evodiamine's potential as an antiviral agent, a property that aligns well with the focus of our study. Moreover, evodiamine has been considered a potential medication option for combating liver diseases (Li et al., 2020). However, to the best of our knowledge, there has been no investigation performed to assess the potentiality of evodiamine derivatives to function as a potential medication option for Lassa fever. Harnessing the power of technology, this study aimed to expedite the discovery process while conserving valuable time, resources, and funding required for developing novel therapeutic options for combating the Lassa virus (Rahman et al., 2012). Several evodiamine derivatives were subjected to computational investigation through systematic approaches, including molecular docking, drug-likeness assessment, molecular dynamics simulation, and ADMET analysis. This theoretical study will add a new dimension in considering these evodiamine derivatives as potential treatment options for treating Lassa fever.

## 2. Computational method

### 2.1. Determination of the data of ADMET

Many drug-like molecules are eliminated from trial phases for not having proper absorption, distribution, metabolism, excretion, and toxicity (ADMET) profile (Alqahtani, 2017). Therefore, we have calculated ADMET profiles for selected evodiamine derivatives, employing the pkCSM (https://biosig.lab.uq.edu.au/pkcsm/prediction) web server (Pires et al., 2015). The pkCSM web server uses a cutting-edge method based on graph-based signatures to predict various pharmacokinetic features. Predictive models may be effectively trained using these signatures for several different ADMET features. The method, known as pkCSM, also offers a platform for the analysis and optimization of pharmacokinetic and toxicity properties implemented in a friendly, open-source web interface, a useful tool to assist medicinal chemists in striking a balance between potency, safety, and pharmacokinetic properties. Assessing the ADMET profiles for selected compounds using this server will help us to select the most suitable compounds that have good absorption, distribution through blood, good metabolic profile, better excretion rate, and lowest toxicity. The pkCSM server accepts SMILES as input; hence, canonical SMILES for compounds containing no chiral carbons and isomeric SMILES for compounds containing chiral carbons were used to generate desired ADMET values.

### 2.2. Preparation of ligand and molecular optimization

Before starting molecular docking, we optimized the three-dimensional structures of the selected compounds using the Materials Studio 8.0 software package (Sharma et al., 2019). This program effectively optimizes the overall geometry and chemical structure of ligands to achieve minimum ground-state energy so that these structures could be docked with receptor proteins without any interruption. After importing the ligand structures into the Material Studio 8.0 software, density-functional theory (DFT) was incorporated by applying the DND basis (diffused basis set) semi-core pseudo-potentials (Papajak and Truhlar, 2010; Obot et al., 2015; Ribeiro et al., 2015). In material research, DFT is commonly used to investigate electronic structure organization using a quantum mechanical modeling strategy. Finally, the structures are saved in a PDB format for further computational analysis.

### 2.3. Preparation of protein and molecular docking studies

**Figure 2:**
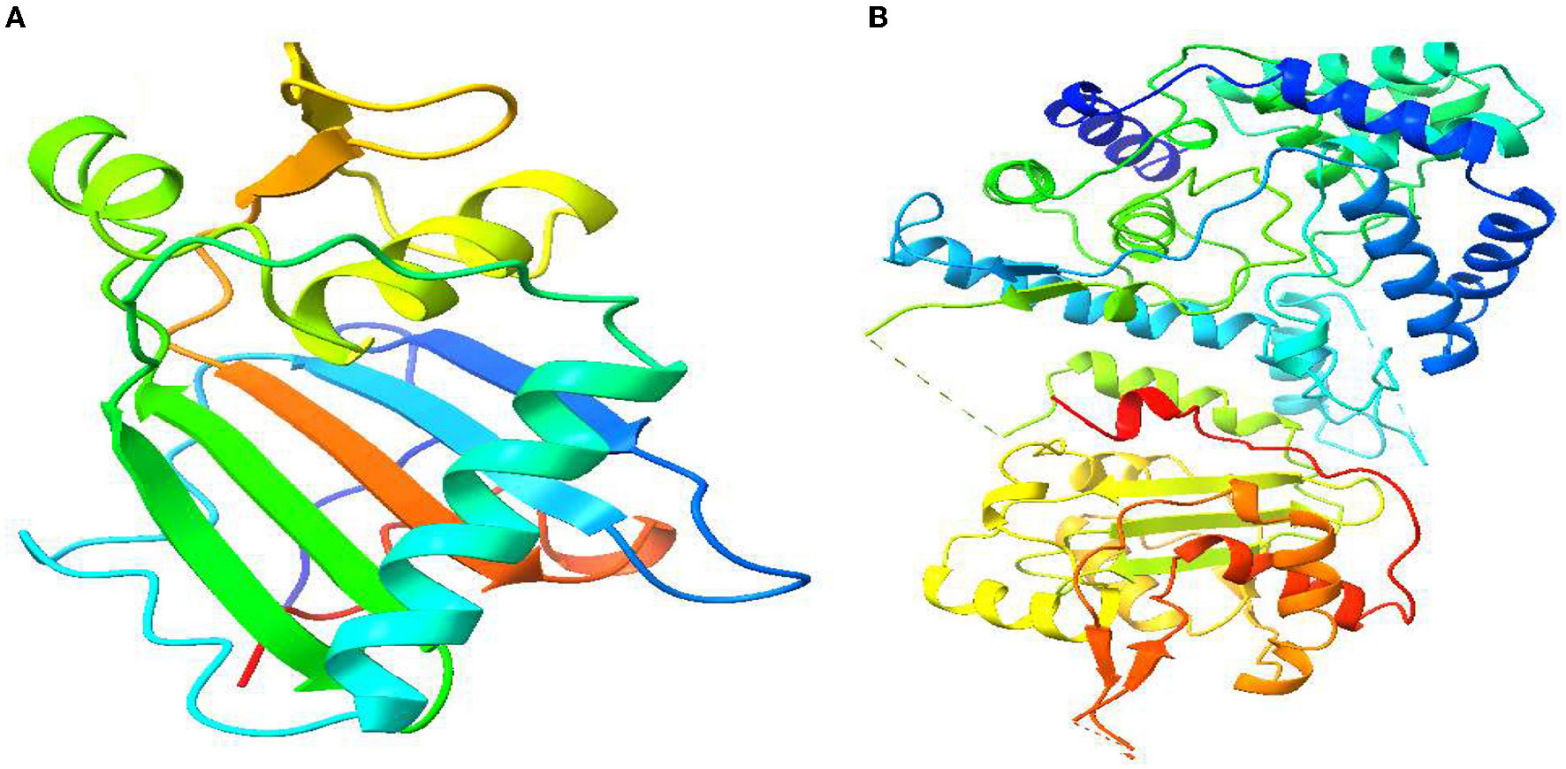
Three-dimensional protein structure of Lassa virus and its basic features. **(A)** Lassa virus glycoprotein spike (PDB ID: 5FT2). **(B)** Lassa virus nucleoprotein (PDB ID: 3MX5).

Molecular docking analysis is a significant aspect of computational drug design (Jakhar et al., 2020). Therefore, in our study, we have incorporated molecular docking analysis to understand the molecular binding dynamics between selected compounds and selected proteins. First, the three-dimensional structures of the Lassa virus glycoprotein spike (PDB ID 5FT2) and Lassa virus nucleoprotein (PDB ID: 3MX5) were acquired from the RCSB protein data bank in the PDB format (Rose et al., 2016). Users of this platform have access to approximately 200,000 experimentally established PDB structures of biological macromolecules and almost a million computed structure models. The PDB structures (Figure 2) were subjected to energy minimization using Swiss PDB Viewer v4.1.0 (Kaplan and Littlejohn, 2001). After energy minimization, both protein structures were opened in BIOVIA Discovery Studio Visualizer to delete excessive water molecules, surrounding the protein which could interrupt ligand–protein docking (Design, 2014). It will be any unwanted heteroatom attached with protein structure were also deleted, concerning these ligands that could occupy/interfere the ligand protein binding. Water molecules surrounding the macromolecules were also eliminated as they do not play a role in ligand–protein molecular interaction. After completing the protein preparation part, we acquired ligand structure files from the PubChem database in SDF format (Kim et al., 2019). The ligand structures were also energy minimized before molecular docking is started. For molecular docking, we have used AutoDock Vina in PyRx software where the ligand structures used were converted into the PDBQT format, and the grid center points were set to X = −21.6734, Y = −17.1276, and Z = 28.1838 and the box dimensions (Å) X = 47.70441, Y = 64.6983, and Z = 51.6696 for (PDB ID: 3MX5), and the grid center for (PDB ID: 5FT2) were set to X = −8.7259, Y = −31.6119, and Z = −27.5813 and the box dimension X = 81.3565, Y = 55.5874, and Z = 91.9504 (Trott and Olson, 2010). PyRx software presents the 9 most suitable docking poses of the ligand–protein complex after the docking is completed (Dallakyan and Olson, 2015). We have selected the most suitable docking poses where the ligands are strongly interacting with the protein's catalytic cavity and visualized them using BIOVIA Discovery Studio Visualizer to have a great insight into ligand binding position in the protein cavity.

### 2.4. Lipinski rule, pharmacokinetics, and drug-likeness

Determination of pharmacokinetic properties is an effective approach to distinguish between drug-like and non-drug-like small molecules. We used the SwissADME server to calculate important drug-like features such as molecular weight, hydrogen bond acceptor, hydrogen bond donor, molar refractivity, topological surface area, and bioavailability (Daina et al., 2017). All these features were well calculated considering Lipinski's rule of five proposed by Lipinski (2004). Distinguishing drug-like molecules by considering Lipinski's rule of five is a globally acceptable approach proposed by Chris Lipinski suggested that any drug-like molecules should follow at least three of the following four rules: (1) A drug-like molecule must have a maximum molecular weight of 500 g/mol or less. (2) The lipophilicity of any drug-like molecules should not cross 5 logP. (3) The maximum number of hydrogen bond acceptors in the drug-like molecule should not cross 10. (4) The maximum number of hydrogen bond donors present in the chemical structure of a drug-like molecule should not cross five. In our investigation, the canonical SMILES of elected evodiamine derivatives and the standard drug sofosbuvir were collected from the PubChem database. The canonical SMILES were inputted into the SwissADME server to calculate selected parameters for the proposed small molecules. The Lipinski rule of five following and violating decisions of selected derivatives was also obtained from SwissADME.

### 2.5. System preparation

The compounds were parameterized by a general AMBER force field (GAFF) for organic molecules with the ANTECHAMBER module implemented by AMBER. Protein was parametrized by AMBER ff14SB force field. The ligands were bound to Lassa fever virus nucleoprotein (PDB ID: 3MX5) in aqueous solutions with an explicit solvent TIP3P water box. NaCl ions were modeled to neutralize the system. The tutorial for the LEaP program was used for the formation of the protein–ligand complexes and the preparation of the system (Case et al., 2005; Shukla and Tripathi, 2020).

### 2.6. Molecular dynamics simulation

The simulations were well carried out by AMBER 16 using the Particle Mesh Ewald (PME) method in each system (Essmann et al., 1995). The systems were prepared as described above. The SHAKE algorithm was used to constrain hydrogen bonds, allowing the use of an integration time of 2 fs. The following molecular dynamics protocol was used: (I) solvent minimization (30,000 steps), (II) equilibrium to heat the system from 100K to 298K at a constant volume with restricted proteins (1 ns), (III) equilibrium to relax the system with restricted proteins (1 ns), (IV) relaxation of the system for 1 ns at constant pressure and 298 K with restriction of less than 10 kcal/mol-Å2 of the protein, (V) minimization of the system with restrictions only on the protein backbone, (VI) relaxation of the system for 1 ns at constant pressure and 298 K with a restriction of <10 kcal/mol-Å2 of the backbone, and (VII) three equilibration steps where the restraint on the backbone was decreased until it was free. Finally, 140 ns of molecular dynamics production was launched.

## 3. Result and discussions

### 3.1. Lipinski rule, pharmacokinetics, and drug-likeness

Evodiamine derivatives are well known for their effective application in different disease treatments, such as pulmonary hypertension, gastric cancer, and hepatocellular carcinoma (Zhang et al., 2020; Fan et al., 2021; Liang et al., 2022). Recently, computational drug designing application of evodiamine derivatives was noticed in the potential treatment of viral diseases such as COVID-19 caused by the SARS-CoV-2 virus (Belal et al., 2022). On that account, we have investigated the pharmacokinetics and drug-likeness properties (Table 1) of our selected evodiamine derivatives to select the most potent small molecule for Lassa fever treatment caused by the Lassa virus. The pharmacokinetic profile of the standard drug sofosbuvir was also calculated for the following comparative analysis. According to SwissADME, all nine of our selected compounds followed the Lipinski rule of five, whereas the compounds named 49806624, 49806625, and 49804912 expressed only one violation of the Lipinski rules. In contrast, the compounds named evodiamine, 49806754, 49806500, 129710532, 151289, and 56967508 all followed Lipinski rules, which prove their credibility to be selected as potential drug candidates for having appropriate pharmacokinetics and drug profile. The calculation of topological polar surface area (TPSA) is a crucial indicator to understand the ability of drug molecule transportation (Ertl et al., 2000). Ideally, a TPSA score of <130 Å^2^ indicates excellent drug-transporting ability in the host system where the minimum TPSA score of a drug-like molecule should not be <20 Å^2^. Interestingly, the standard drug sofosbuvir has a TPSA score of 167.99 Å^2^ which crosses the ideal range of TPSA. All nine selected evodiamine compounds had a TPSA score of <130 Å^2^, indicating their excellent transporting ability as a drug in the host system. All nine selected evodiamine compounds expressed a bioavailability score of 0.55, which was significantly higher than sofosbuvir. The lipophilicity value was also calculated to predict non-aqueous solubility. By assessing the data presented, we predict that compound 129710532 showed the lowest score (2.44 Log Po/w) and compound 49806624 showed the highest lipophilicity score (4.54 Log Po/w). For an ideal drug-like molecule, the molar refractivity score should be between 40 and 130 units. Only three compounds (49806624, 49806625, and 49806500) had slightly higher scores than the ideal range. However, the molar refractivity scores for the other six compounds were satisfying.

**Table 1:** Data of Lipinski rule, pharmacokinetics, and drug-likeness.

**Sl. No**.	**CID**	**Molecular weight (g/mol)**	**Consensus Log Po/w**	**Hydrogen bond acceptor**	**Hydrogen bond donor**	**Molar refractivity**	**Topological polar surface area Å^2^**	**Lipinski rule**		**Bioavailability Score**
								**Result**	**Violation**	
01	Evodiamine	303.36	2.7	1	1	97.67	39.34	Yes	0	0.55
02	49806754	342.39	2.52	2	0	107.13	52.27	Yes	0	0.55
03	49806624	427.93	4.54	1	0	132.07	28.48	Yes	1	0.55
04	49806625	427.93	4.48	1	0	132.07	28.48	Yes	1	0.55
05	49806500	437.49	3.76	3	0	133.97	54.78	Yes	0	0.55
06	129710532	321.37	2.44	2	2	100.72	48.57	Yes	0	0.55
07	151289	303.36	2.7	1	1	97.67	39.34	Yes	0	0.55
08	56967508	289.37	3	1	1	97.25	22.27	Yes	0	0.55
09	49804912	425.45	4.03	3	0	127.44	45.55	Yes	1	0.55
**Standard:** Sofosbuvir		529.45	1.44	11	3	125.53	167.99	No	2	0.17

### 3.2. Molecular docking and interaction analysis

Lassa virus glycoprotein spike (PDB ID: 5FT2) and Lassa virus nucleoprotein (PDB ID 3MX5) were docked with evodiamine's nine selected derivatives. These docked complexes were compared with the standard drug sofosbuvir for justifying the significance of conducting this study. According to the molecular docking rules, a stable protein–ligand complex should express minimum binding energy with a strong binding affinity of the ligand with the receptor protein. Sofosbuvir showed binding energy of −5.9 kcal/mol for the Lassa virus glycoprotein spike. However, eight (ligand nos: 01, 02, 03, 04, 05, 07, 08, and 09) out of nine selected evodiamine derivatives expressed higher binding affinity than the standard drug. These docking scores indicate that ligand nos. 03, 04, and 09 have bound much more strongly than sofosbuvir with the Lassa virus glycoprotein spike. As stronger binding has a positive correlation with forming a more stable receptor-ligand complex, we can suppose that our selected compounds will have a better role in stabilizing the target protein than the stronger drug.

In addition, Lassa virus nucleoprotein was also docked with the same ligands. Sofosbuvir showed a binding energy score of −7.1 kcal/mol with this receptor. Except for ligand no. 06, all eight ligands showed better docking scores than the standard drug (Table 2). Ligand no. 04 had a binding energy score of only −11 kcal/mol, indicating excellent binding with the receptor by forming a stable protein–ligand complex. Moreover, ligand nos. 01, 03, 05, 07, and 09 also showed excellent binding affinity, suggesting that they could be also considered for potential future drug development. After analyzing all the docked complexes, it could be said that ligand nos. 04 and 09 expressed excellent docking scores with both target receptors, which was much higher than sofosbuvir. These two compounds can be considered very strong candidates for developing future drug development against Lassa Virus.

**Table 2:** Molecular docking/binding energy score for all derivatives represented.

**Sl. No**.	**PubChem CID**	**Chemical structure**	**Lassa virus glycoprotein spike (PDB ID 5FT2)**	**Lassa virus nucleoprotein (PDB ID 3MX5)**
			**Binding affinity (kcal/mol)**	**Binding affinity (kcal/mol)**
01	Evodiamine	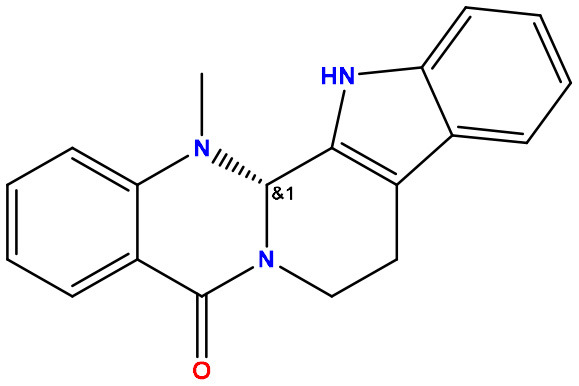	−7.2	−10.7
02	49806754	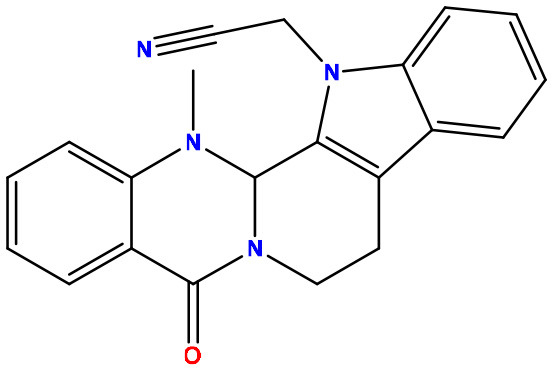	−7.4	−9.1
03	49806624	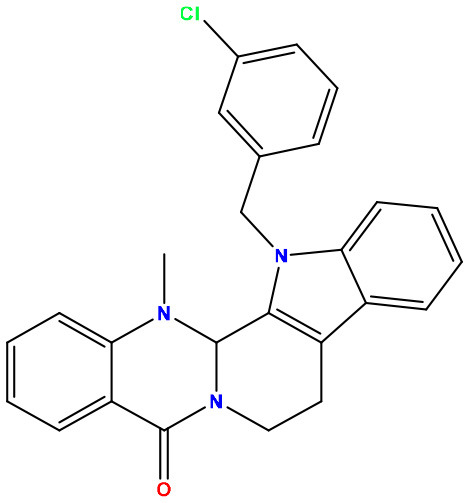	−8.3	−9.7
04	49806625	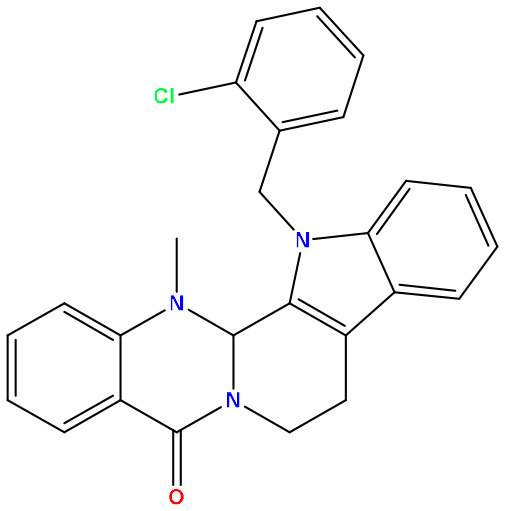	−8.5	−11.0
05	49806500	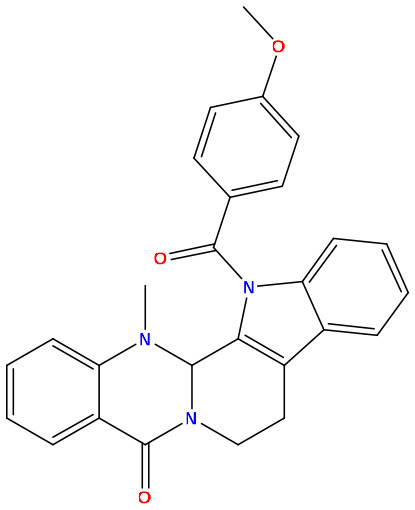	−8.1	−9.8
06	129710532	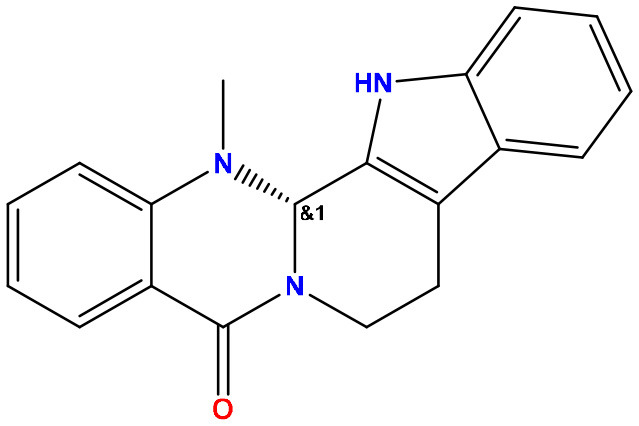	−1.7	−2.0
07	151289	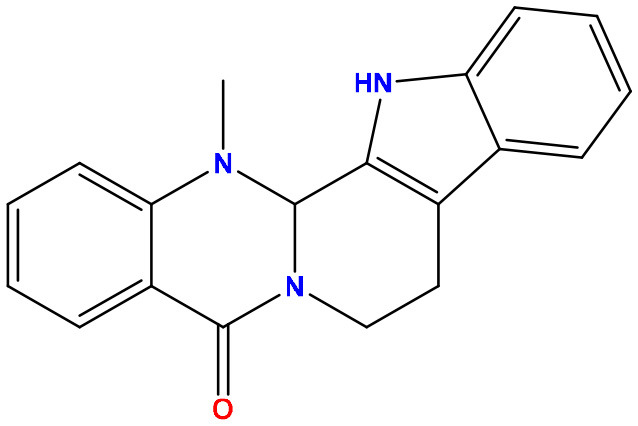	−7.8	−10.6
08	56967508	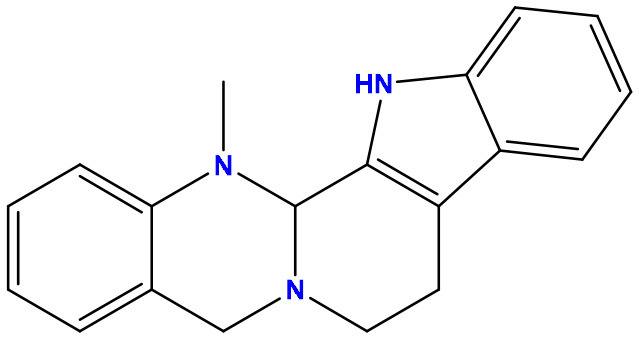	−7.2	−9.4
09	49804912	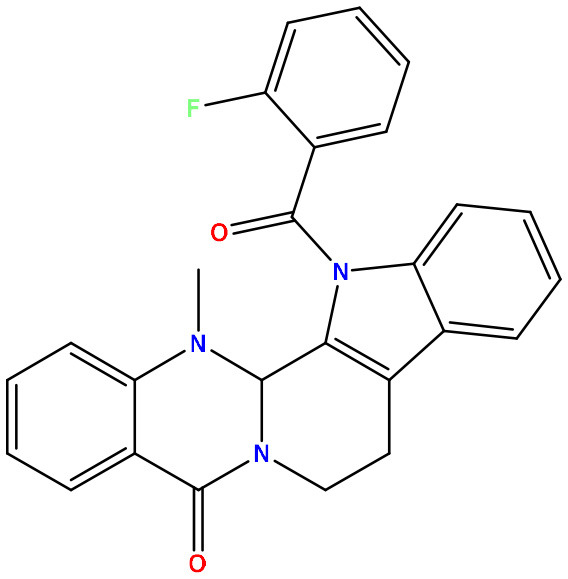	−8.3	−10.3
Standard Sofosbuvir		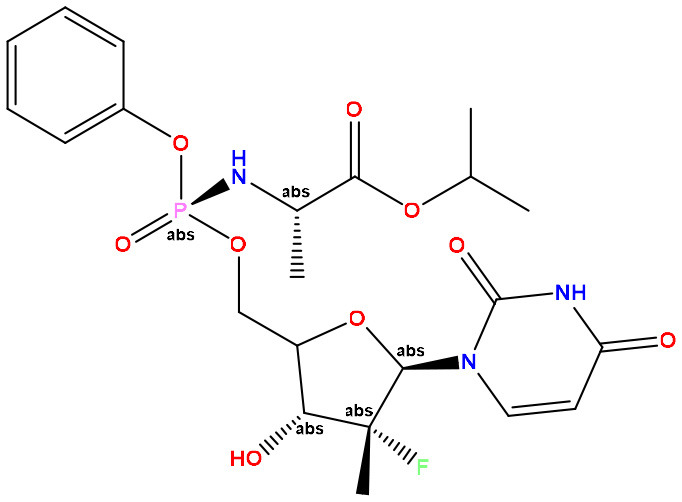	−5.9	−7.1

### 3.3. Protein–ligand interaction and molecular docking poses

**Figure 3:**
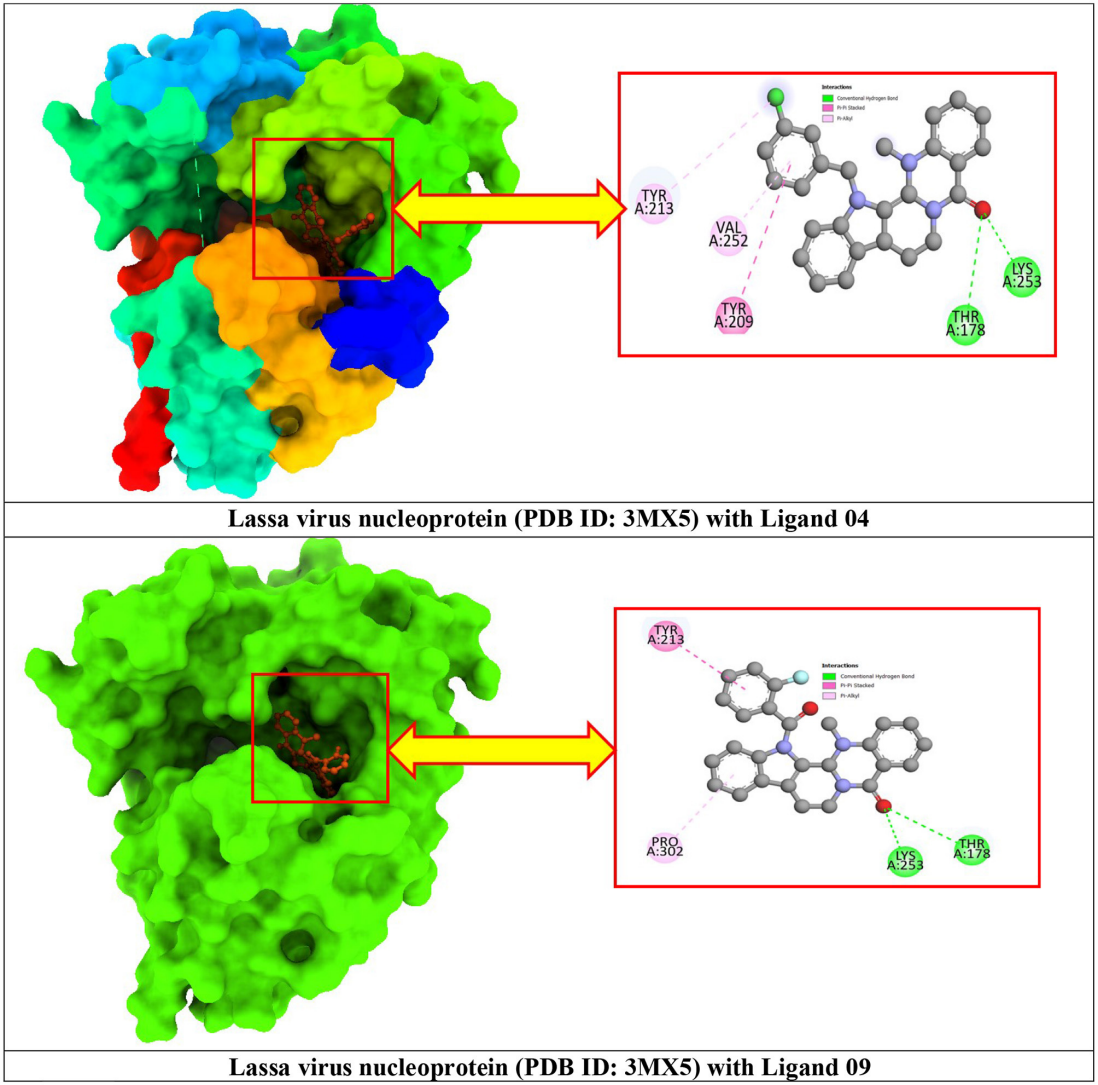
Docking interactions between the proposed compounds.

Protein–ligand docked complexes were visualized using the PyMOL program to better understand different types of interactions. For the Lassa virus nucleoprotein (PDB ID: 3MX5), compound nos. 04 (−8.5 kcal/mol) and 09 (−8.3 kcal/mol) had the maximum binding energy. Compound no. 04 formed a Py-Alkyl bond with TYR A:213 and VAL A:252; a conventional hydrogen bond with THR A:178 and LYS A:253; and pi-pi stacked with TYR A:209 (Figure 3). However, compound no. 09 formed a Pi-Alkyl bond with PRO A:302; a conventional hydrogen bond with LYS A:253 and THR A:178; and pi-pi stacked with TYR A:213 (Figure 3). Selected protein ligands with strong molecular interactions were further analyzed by incorporating molecular dynamics simulation to explore the significant roles of selected compounds in stabilizing virulent proteins.

### 3.4. Molecular dynamics simulation analysis

The production of the molecular trajectory (140 ns) was used to perform the analyses. The root mean square deviation (RMSD) allows one to calculate the divergence between the two overlapping structures, so the lower the value, the higher the similarity between them. The RMSD and RMSF plots were performed using Visual Molecular Dynamics (VMD) (Humphrey et al., 1996a,b). VMD and TCL scripts to establish residues that have contact with the ligand at a distance of fewer than 5 Å for at least 50% of the molecular trajectory. The binding free energy calculations for the protein–ligand systems were estimated using the Python script MMPBSA.py provided by AMBER.

**Figure 4:**
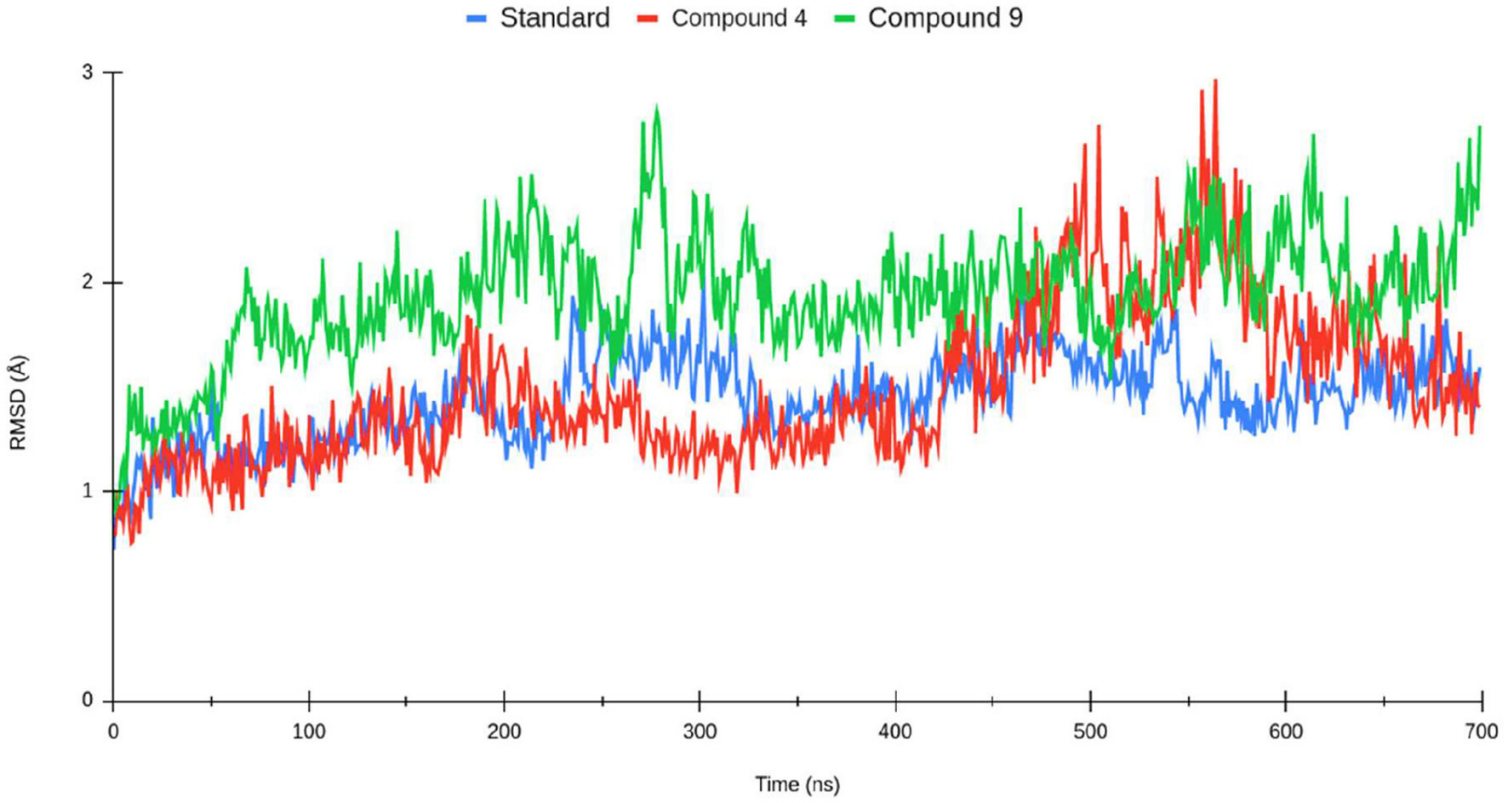
Root mean square deviation (RMSD) of the Lassa virus nucleoprotein (PDB ID: 3MX5) backbone for standard sofosbuvir complex (blue), compound 1 (red), and compound 2 (green).

The root mean square deviation (RMSD) was calculated for the systems described above (shown in Figure 4). The protein–ligand complexes were equilibrated at 40 ns from the molecular dynamics trajectory. In the standard complex, the RMSD had values of 1~2 Å. The complexes formed by compounds 1 and 2 reached higher RMSD values. Compound 4 had fluctuations of 1~3 Å; at the 100th nanosecond of molecular dynamics, the protein rotates sharply, which twists the N-terminus and C-terminus of the proteins, justifying the increase of the RMSD value. Compound 2 achieved similar RMSD values as compound 1; however, compound 2 is more stable along the molecular dynamic trajectory.

**Figure 5:**
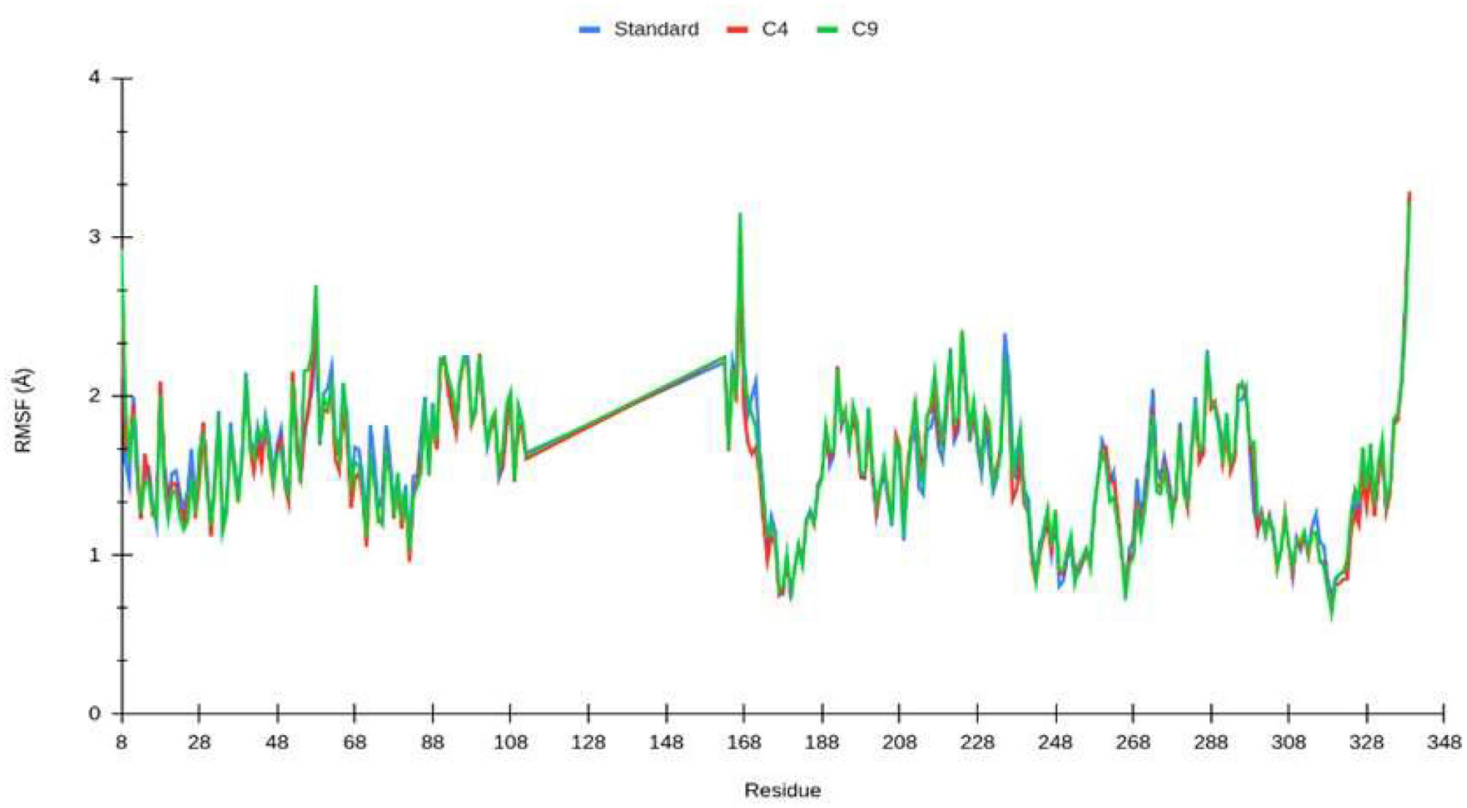
Root mean square fluctuation (RMSF) of Lassa fever nucleoprotein backbone over time (140 ns) for standard (blue), compound 4 (red), and compound 9 (green). Residues 113 to 162 are not present in the nucleoprotein structure.

To characterize the local changes in the interaction motifs close to the ligand, the root mean square fluctuation (RMSF) between the nucleoprotein and the ligand was calculated (Figure 5). The RMSF values are similar in all systems. The most stable interaction correspond to the residues that are found to keep interacting with the ligand. The most stable interaction motifs are located near the N-terminus (8–50 aa.) and in the region between residues 180 and 200, corresponding to the amino acids found to have contact with the ligand.

From the molecular dynamics simulation, we estimated the residues that possessed ligand–ligand contacts at 5 Å for at least 50% of the molecular dynamics. We found 14, 13, and 16 protein–ligand contacts for the standard, compound 4, and compound 9 systems, respectively (Figure 5). Common residues TYR: A 209, TYR: A 206, SER: A 238, LEU: A 239, SER: A 247, LEU: A 248, and GLY: A 249 were found forming contacts in the systems. Among these, the GLY: A 249 residue has a prominent role, forming contacts in all systems in at least 95% of the molecular dynamics trajectory.

The free energy of the protein–ligand binding was calculated by the MMGBSA method to estimate which compound binds better to the nucleoprotein. Table 3 shows the union energy residues for each system. A comparison of the binding free energy values shows that Ribavirin is less stable than compounds 4 and 9. From the above analysis, compound 9 was chosen because it has a higher number of contacts with the nucleoprotein and has a favorable binding energy compared with the other ligands.

**Table 3:** Binding free energy calculations for the protein–ligand systems based on MMGBSA.

**MMGBSA**	**Delta G**	**Standard. dev**.
Standard	−29,4833	±6,1179
Compound 01	−39,4656	±7,8665
Compound 09 09	−36,3851	±3,7805

Values expressed in kcal/mol.

**Figure 6:**
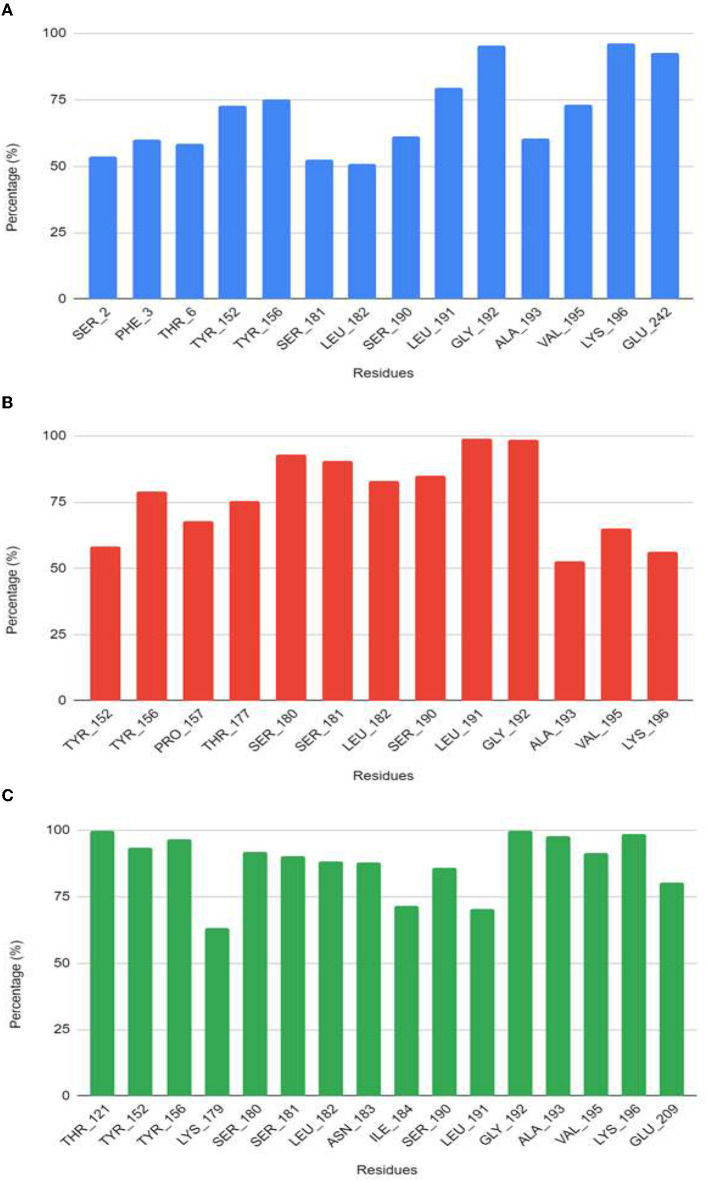
Residues that have contacts at <5 Å with the ligand for at least 50% of the molecular dynamics trajectory for the systems **(A)** sofosbuvir, **(B)** compound 4, and **(C)** compound 9.

**Figure 7:**
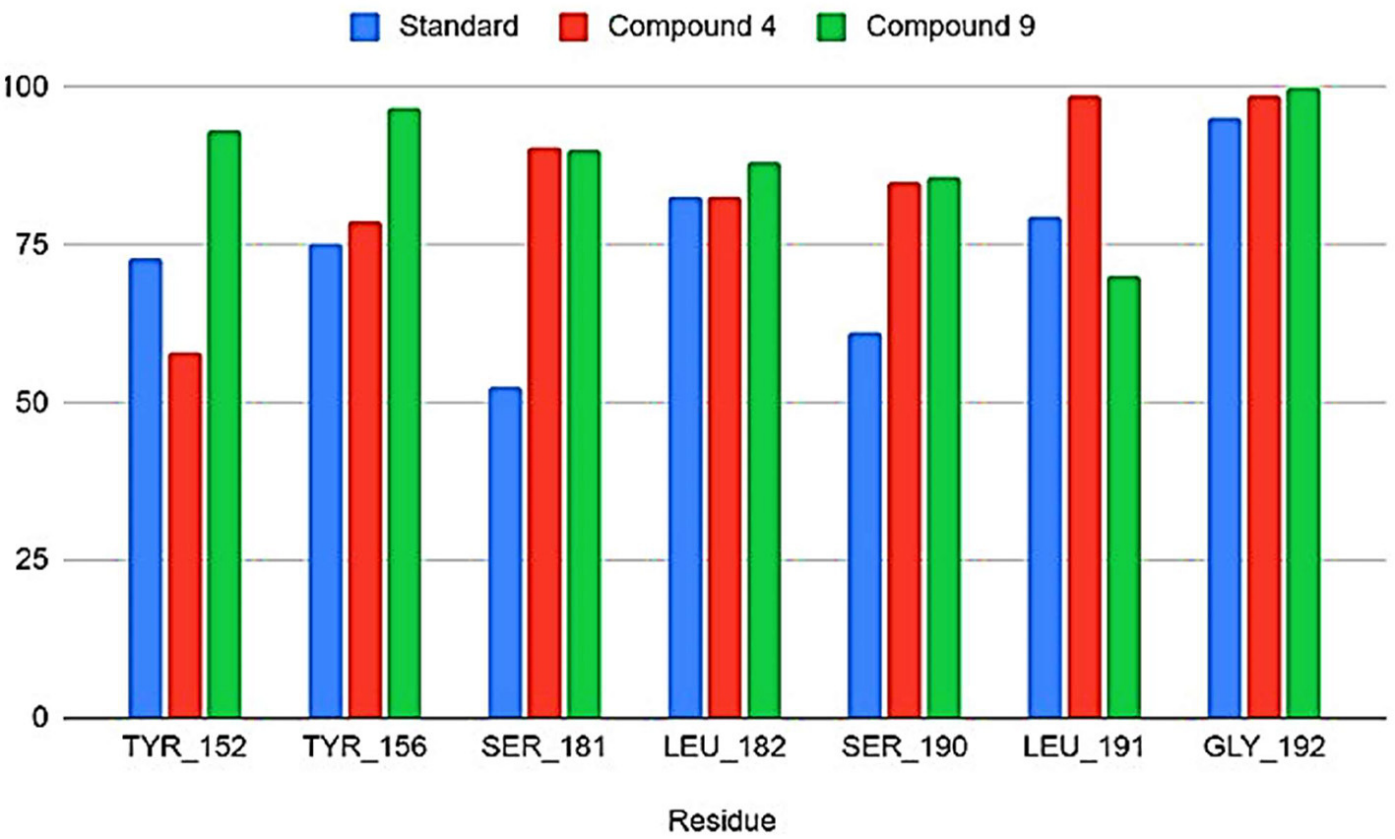
Common residues among contacts found.

The free energy of the protein–ligand binding was calculated by the MMGBSA method to estimate which compound binds better to the nucleoprotein. Figures 6, 7 show the union energy residues for each system. A comparison of the binding free energy values shows that sofosbuvir is less stable than compounds 4 and 9. From the above analysis, compound 9 was chosen because it has a higher number of contacts with the nucleoprotein and has a favorable binding energy compared with the other ligands.

### 3.5. ADMET data investigation

The consumption of drugs of poor ADMET properties can lead to side effects such as allergic reactions, rashes, and organ damage. To avoid these complications, we have calculated the most important ADMET properties for our selected evodiamine derivatives (Table 4). To understand the absorption aspect, we have selected the following three parameters: water solubility, Caco-2 permeability, and human intestinal absorption. According to the data collected from the pkCSM server, the standard drug sofosbuvir has a low human intestine absorption rate of only 60.168%. In contrast to that, all our proposed compounds have higher human interest in absorption rate where compound 05 showed the highest score of 96.562%. According to the water solubility test (calculated in Log S), the range from highly soluble compounds to insoluble compounds is <-10 poorly <- 6, moderately <-4 soluble <-2 very <0 <highly. As presented in Table 4, compounds 01, 06, 07, and 08 were declared as soluble. Furthermore, compounds 02, 03, 04, 05, and 09 were declared as moderately soluble. The Caco-2 cell line, which is normally utilized as a reliable model to evaluate the absorption property of any orally delivered medications, is made up of human colorectal adenocarcinoma epithelial cells (Sambuy et al., 2005). According to pkCSM guidelines, a Caco-2 permeability score over 0.9 is considered high, and the standard drug sofosbuvir expressed a low Caco-2 permeability score of 0.454. All nine proposed compounds showed a higher Caco-2 permeability score than the sofosbuvir, where compound 08 showed the highest score of 1.519.

**Table 4:** Theoretical ADMET data analysis.

**Sl. No**.	**Absorption**			**Distribution**		**Metabolism**		**Excretion**		**Toxicity**		
	**Water solubility Log S**	**Caco-2 permeability** × **10**^−6^	**Human intestinal absorption (%)**	**VDss (human)**	**BBB permeability**	**CYP450 1A2 inhibitor**	**CYP450 2D6 substrate**	**Total clearance (ml/min/kg)**	**Renal OCT2 substrate**	**Max. tolerated the dose (log mg/kg/day)**	**Skin sensitization**	**Hepatotoxicity**
01	−3.71	1.226	93.70	0.327	0.470	Yes	No	0.355	No	0.051	No	Yes
02	−4.193	1.08	95.43	0.402	0.274	Yes	No	0.468	No	−0.436	No	Yes
03	−4.881	1.054	94.24	0.371	0.437	Yes	No	0.156	No	0.473	No	Yes
04	−4.807	1.053	94.53	0.378	0.424	Yes	No	0.217	No	0.467	No	Yes
05	−5.064	1.3	96.56	−0.243	−0.013	Yes	No	0.213	No	0.193	No	Yes
06	−3.609	0.882	94.96	0.188	0.398	Yes	No	0.360	No	−0.046	No	Yes
07	−3.71	1.226	93.70	0.327	0.470	Yes	No	0.355	No	0.051	No	Yes
08	−3.607	1.519	93.27	0.915	0.373	Yes	Yes	1.123	No	0.276	No	Yes
09	−4.421	1.147	96.34	−0.134	0.173	Yes	No	0.132	No	0.514	No	Yes
10	−3.836	0.454	60.16	−0.744	−1.876	No	No	−0.117	No	0.568	No	Yes

For predicting the distribution, we have selected the volume of distribution of the stead-state method (VDss) and blood–brain barrier (BBB) as our keynote parameters. The VDss values indicate how evenly drugs are distributed between the blood and the tissue. A higher VD score (>0.45) suggests that the therapeutic molecule is dispersed more evenly throughout the body, whereas a lower (< -0.15) result denotes uneven drug distribution. Compound number 08 showed excellent VDss distribution, and other derivatives also expressed satisfying VDss scores, except for compound 05. The BBB shields our brain from any interactions with outside substances. This implies that BBB permeability assessment is a crucial characteristic in choosing ideal drug-like compounds (Passeleu-Le Bourdonnec et al., 2013). When the BBB permeability score is <-1, the distribution is poor. In contrast, a score of >0.3 indicates excellent BBB permeability. Compounds 01, 03, 04, 06, 07, and 08 showed positive BBB permeability, whereas compounds 02, 05, and 09 showed poor blood–brain permeability. In metabolic profile analysis, it can be stated that all selected compounds showed positive CYP450 1A2 inhibition, and none of the compounds inhibited the CYP450 2D6 substrate.

Estimating overall clearance and organic cation transporter 2 (OCT2) allowed for the incorporation of excretion analysis (Filipski et al., 2009). Total clearance produces a total clearance score using the combined information from hepatic clearance and renal clearance, which provides a clear excretion profile of any particular drug. All selected derivatives showed better total clearance scores than sofosbuvir. None of the compounds were predicted to be potential renal OCT2 substrates. Finally, in toxicity prediction, we have predicted that none of the substances induce skin sensitization, and special precautions should be taken before recommending these compounds to patients suffering from liver diseases, as all of the selected compounds can induce hepatotoxicity including sofosbuvir.

## 4. Harnessing evodiamine derivatives for lassa virus intervention—a thought-provoking discussion

In the subsequent study, Lassa virus nucleoprotein and Lassa virus glycoprotein spike were considered potential drug targets to inhibit the Lassa virus. *Evodia rutaecarpa* is a rich source of evodiamine, an alkaloid that has garnered scientific interest for its potent therapeutic effects against various diseases such as anti-obesity, anti-allergenic, analgesic, and anti-ulcerogenic properties (Wang et al., 2008; Tan and Zhang, 2016). Furthermore, in the hippocampus, evodiamine significantly reduces neuroinflammation (TNF-α, IL-1β, and IL-6) and glial cell activation, rendering it a potential treatment for neurodegenerative diseases such as Alzheimer's disease (Wang et al., 2018). Despite concerns about evodiamine-induced hepatotoxicity and cardiotoxicity, its effectiveness against various cancer cells (lung cancer, gastric cancer, oral cancer, colorectal cancer, and pancreatic cancer) cannot be dismissed (Wei et al., 2012; Sachita et al., 2015; Wen et al., 2015; Zhao et al., 2015; Zou et al., 2015; Yang W. et al., 2017). Additionally, evodiamine exhibits anti-inflammatory and antioxidative stress potency, and one study suggests its potential as a therapeutic lead compound in liver diseases (Zhang et al., 2018, 2022; Li et al., 2020).

Given the broad-spectrum antiviral effects of alkaloids against various DNA and RNA viruses, evodiamine has also demonstrated promising therapeutic effects against viruses such as influenza A virus (Dai et al., 2012; Abookleesh et al., 2022). Consequently, natural evodiamine derivatives were considered for identifying potential therapeutic agents for Lassa fever treatment in the present study. In light of the comprehensive findings, the ingeniously designed evodiamine demonstrates promising potential in combating both the Lassa virus glycoprotein spike and the Lassa virus nucleoprotein, with favorable ADMET and drug-likeness properties and molecular dynamic simulations validating their stability.

## 5. Conclusion

Lassa fever, a neglected tropical disease, lacks FDA-approved vaccines and has limited treatment options. This study aimed to identify promising therapeutic candidates, focusing on evodiamine derivatives from the PubChem database. Using the PyRx application, computational docking was performed, followed by theoretical bioavailability and toxicological predictions via pkCSM and Swiss ADME tools. The results revealed that most selected inhibitors demonstrated favorable binding energies with the Lassa virus glycoprotein spike and nucleoprotein. Molecular dynamic simulations supported the stability of the protein–ligand complexes. Meeting Lipinski's criteria, ADME analysis indicated that the investigated inhibitors were generally safe. However, patients with impaired liver function should exercise caution due to potential hepatotoxicity. Overall, evodiamine derivatives showed potential as inhibitors against Lassa virus glycoprotein spike and nucleoprotein, warranting further wet lab validation to confirm these *in silico* findings.

## Data availability statement

The datasets presented in this study can be found in online repositories. The names of the repository/repositories and accession number (s) can be found in the article/supplementary material.

## Author contributions

SA and NM: conceptualization, writing original draft, and analysis. JB, SM, NM, VS, MI, and GG: initial draft and methodology. VR, SC, GR, and FS: visualization. GA, AS, and MA-D: revised and edited the manuscript. All authors contributed to the article and approved the submitted version.
